# The International Advanced Practice Nurse Integration Policy Intervention Taxonomy: A 10‐Country Nominal Group Consensus Technique Study

**DOI:** 10.1111/inr.70096

**Published:** 2025-08-12

**Authors:** Joshua Porat‐Dahlerbruch, Tatyana Miller, Jocelyn Boyd, Moriah E. Ellen, Rebecca R. S. Clark

**Affiliations:** ^1^ Department of Acute and Tertiary Care School of Nursing University of Pittsburgh Pittsburgh Pennsylvania USA; ^2^ Israel Implementation Science and Policy Engagement Centre Ben‐Gurion University of the Negev Beer‐Sheva Israel; ^3^ Herzog Medical Center Jerusalem Israel; ^4^ Department of Health Policy and Management Faculty of Health Sciences and Guilford Glazer Faculty of Business Ben‐Gurion University of the Negev Beer‐Sheva Israel; ^5^ Center for Health Outcomes and Policy Research School of Nursing University of Pennsylvania Philadelphia Pennsylvania USA; ^6^ Pennsylvania Hospital Philadelphia Pennsylvania USA

**Keywords:** advanced practice nursing, consensus, implementation, international nurse, nurse practitioner, nursing model

## Abstract

**Aim:**

To develop an international taxonomy of policy interventions for integrating advanced practice nurses into health systems.

**Background:**

Advanced practice nurse integration is the extent to which advanced practice nurses can function to the full extent of their education and scope of practice and contribute to better outcomes. Several barriers to integration are common across countries, such as an ill‐defined scope of practice, lacking leadership engagement, and insufficient knowledge of the role. Barriers are attributed to a dearth of knowledge on evidence‐based policy interventions guiding integration.

**Methods:**

This was a nominal group expert consensus study with participants from multiple countries. Participants were divided into three meetings consisting of silent review of a preliminary taxonomy developed from a literature review and single‐country testing, round‐robin feedback, live revisions, and consensus voting. Each meeting resulted in a unique taxonomy, which researchers synthesized into one version. The synthesized taxonomy was sent for member checking and final consensus voting. The Accurate Consensus Reporting Document was used.

**Results:**

There were 12 experts representing 10 countries. Fifty‐nine policy interventions were mapped to 14 categories. National‐level categories included regulation, economic incentives, stakeholder cooperation, education and workforce development, marketing, and research. Organizational interventions included organizational guidelines, infrastructure development and resource allocation, interprofessional leadership engagement, and organizational messaging. Care team–level interventions included interprofessional experience/exposure, team communication, work environment, and research opportunities.

**Discussion:**

This taxonomy builds on prior research by including participants across countries with varying health system governance, wealth, and advanced practice development.

**Conclusion:**

Applying this taxonomy may contribute to more efficacious advanced practice nursing integration and potentially improve outcomes.

**Implications:**

A guide for adapting interventions to a health system, organization, or care team is included.

## 1 Introduction

Health systems worldwide are facing increasing pressure to address the growing demand for healthcare services (World Health Organization 2025). Introducing, developing, and deploying advanced practice nurse (APN) roles is one solution to meet demand for healthcare service delivery (International Council of Nurses 2020). The International Council of Nurses (2020, p. 6) describes APNs as “a generalist or specialized nurse who has acquired, through additional graduate education, the expert knowledge base, complex decision‐making skills, and clinical competencies shaped by the context in which they are credentialed to practice.” APNs with prescribing authorities, which are the focus of this article, are of particular interest to health systems for two reasons. First, APNs who prescribe can fill provider shortages (Maier et al. 2017). Second, international evidence indicates that APNs provide patient‐centered, holistic care that accounts for community, family, and individual circumstances shaping health outcomes (Kinchen 2019; Porat‐Dahlerbruch et al. 2022). These positive contributions of APNs drive much of the impetus for introducing APN roles into health systems (Poghosyan et al. 2023).

Realizing these positive outcomes associated with APN care requires health systems to move beyond the *introduction* of APNs by emphasizing the *integration* of APNs (Porat‐Dahlerbruch et al. 2023a). APN integration is defined as “a multi‐level process of incorporating APNs into a care model to the extent that they can function to their full scope of practice and education, which leads to improved patient, health system, and population needs” (Porat‐Dahlerbruch et al. 2025b, 2022). Insufficient research attention has been placed on developing evidence‐based policies enabling the efficacious integration of APNs into health systems (Torrens et al. 2020). However, at national/regional (macro), organizational (meso), and point‐of‐care (micro) levels, health system actors (e.g., policymakers and management) require knowledge of effective evidence‐based policies facilitating the integration of APNs (Porat‐Dahlerbruch et al. 2022). Such knowledge of evidence‐based policies is particularly critical for countries in the early stages of APN integration (e.g., India and Czechia), though it could benefit countries in the more advanced stages of integration (e.g., the United States, Canada, and Australia) still facing hindrances to APN integration, such as limiting autonomy at legislative and organizational levels (Porat‐Dahlerbruch et al. 2023a).

## 2 Background

International research has identified several common barriers to APN integration across countries. Barriers at the macro level include restrictive scope of practice laws and financing schemes insufficiently incentivizing organizations to hire and retain APNs (Maier et al. 2017). Single‐country studies in Israel and India have found that non‐university–based education programs, unclear professional development pathways, and the absence of professional organizations for APNs act as barriers to integration (Andrews et al. 2021; Kodi and Sharma 2021; Porat‐Dahlerbruch et al. 2025a). Reports in the United States and Australia, which have relatively long histories of APNs, found that national physician groups resist expanded APN autonomy because of conceptions that APNs provide poorer care quality than physicians or that APNs are not educated to provide autonomous care (National Academies of Sciences, Engineering, and Medicine 2021; Peters et al. 2024).

Several meso‐ and micro‐level barriers have also been identified. On the meso level, a study in Norway identified insufficient organizational infrastructure and lacking support from executives as hindrances to APN integration (Henni et al. 2019). One review of APN integration in primary care settings reported that organizations do not provide sufficient time for the mentorship necessary for fostering APN professional development (Torrens et al. 2020). On the micro level, APNs working in poorer primary care work environments in the United States are four times more likely to report poorer care quality (Brooks Carthon et al. 2022). Research in New Zealand found that APN practice is restricted by interprofessional colleagues who perceived APNs as “physician extenders” and not an autonomous role (Carryer and Adams 2017). Finally, an international review found that physicians working with APNs often under‐value APN care because of lacking confidence in their educational readiness (Torrens et al. 2020).

Taken together, countries around the world with shorter and longer histories of APNs would benefit from policy guidance to advanced APN integration at macro, meso, and micro levels (Porat‐Dahlerbruch et al. 2023a). Implementing evidence‐based integration policies has been linked to better APN integration and improved patient, population, and system outcomes (Porat‐Dahlerbruch et al. 2025b, 2022).

### 2.1 Study Purpose

Health systems, especially in countries where the APN role is new, often integrate APNs into the workforce without developing comprehensive policies offsetting integration barriers (Porat‐Dahlerbruch et al. 2025c; Schorn et al. 2022). A failure to develop policies offsetting barriers to integration before introducing the role leads to a prolonged and less efficacious integration process—it is difficult to change policies once enacted (Porat‐Dahlerbruch et al. 2025c, 2023b). As such, poor APN integration planning leads to resource waste. This phenomenon has been termed “ad hoc” integration (Bryant‐Lukosius et al. 2004). Many countries, moreover, hesitate to develop APN roles because policy decision‐makers lack knowledge on effective policy development (Maier et al. 2017). Given the widespread lack of evidence‐based knowledge on policy interventions facilitating APN integration, it would be prudent to provide guidance for health system actors.

Scholars have called for the development of a comprehensive set of policy interventions to guide health system actors leading APN integration efforts (Poghosyan et al. 2023; Porat‐Dahlerbruch et al. 2023a). In a seminal review of laws and regulations affecting APN integration in Organization for Economic Cooperation and Development countries, Maier et al. (2017) concluded that the APN integration process is similar across countries. Since then, two international reviews of factors affecting APN integration have reaffirmed this conclusion and added that similarities exist not only at the macro level but also at the meso and micro levels (Porat‐Dahlerbruch et al. 2022; Torrens et al. 2020). Accordingly, we posit that it is possible to develop a comprehensive, global taxonomy of policy interventions to guide APN integration at macro, meso, and micro levels. Similar to implementation science taxonomies and frameworks, health system actors can adopt specific policy options from a global taxonomy that are relevant for their specific context (e.g., nation, jurisdiction, care organization, or unit/clinic) (Damschroder et al. 2022). No such taxonomy, applicable to multiple countries, exists to date (Porat‐Dahlerbruch et al. 2023a). To fill this policy need, this study aimed to develop an international taxonomy of policy interventions for integrating APNs into health systems at macro, meso, and micro levels.

## 3 Methods

### 3.1 Study Design

This qualitative, descriptive study was conducted using a nominal group consensus technique (NGCT). NGCTs are highly structured group interactions that facilitate discussion among experts and conclude in consensus (McMillan et al. 2016). We selected a consensus approach for three reasons aligned with recommendations by Belton et al. (2019). First, there is insufficient research synthesizing APN integration policy interventions applicable to multiple countries. Second, multiple, widely varying perspectives are needed to develop an international taxonomy. Third, the odds that health system actors across multiple countries will use the taxonomy are higher when requiring consensus among a multi‐national group of participants. An NGCT was selected instead of other expert consensus techniques, such as a Delphi study, because live discussion is more efficient and effective for ensuring that the taxonomy applies to multiple countries (Manera et al. 2019). Further, NGCTs promote balanced participation by ensuring that each expert vocalizes thoughts and votes on agreeability. We used the Accurate Consensus Reporting Document as an EQUATOR guideline (Gattrell et al. 2024).

### 3.2 Participants and Recruitment

In expert consensus studies, participants are chosen based on convenience or purposive sampling and should include academic and practice experts (Belton et al. 2019). Academic experts must have published at least three peer‐reviewed, empirical papers on APN integration as a lead investigator (first or senior author) and still be working in academia. Practice experts must have been working as a government policymaker or a large care organization manager responsible for integrating APNs. We aimed to reach the recommended 4–14 participants (Manera 2019).

We used two processes to select participants meeting the inclusion criteria. First, an international advisory panel of five experts (three academic and two practice experts) nominated participants according to our inclusion criteria. We encouraged panelists to consider nominations from multiple countries and regions. Second, we examined a list of articles included in the most recent international literature review on APN integration, which included 78 peer‐reviewed sources (Porat‐Dahlerbruch et al. 2022). The team searched for each lead investigator on the included article list to determine if they met the inclusion criteria. The advisory panel nominated 74 experts with rationale (Belton et al. 2019). After the study team verified whether nominees met the inclusion criteria, 57 nominees were deemed eligible. Our examination of the literature resulted in an additional 12 participants not included in the qualifying nominations, rendering 69 total eligible participants. We sent an e‐mail invitation up to three times, each at two‐week intervals. The e‐mail explained the study aims, ethical considerations, and time commitment. We did not offer compensation.

### 3.3 Data Collection

**FIGURE 1:**
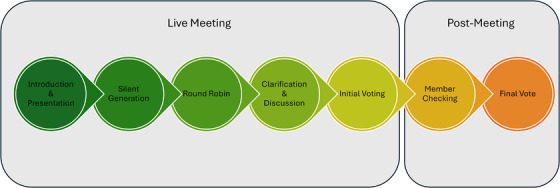
Nominal group consensus technique process. This nominal group consensus study consisted of two phases—live meeting and post‐meeting phases. During the live meeting, there were five steps. First, during the introduction and presentation, we presented the meeting goals and initial taxonomy (Porat‐Dahlerbruch et al., 2024). In the silent generation, the participants had five minutes to review the model and write down their thoughts. During the round‐robin stage, the participants each voiced their key points. In clarification and discussion, the participants participated in open debate and live revisions of the model. Finally, the participants each voted on initial consensus. The post‐meeting phase consisted of member checking, in which the participants received the revised model and provided feedback. A final consensus vote was held for the final model.

An NGCT meeting is typically conducted at one time with all participants. However, the international nature of this study required us to accommodate participants from multiple time zones. Accordingly, we divided the 12 participants into three video‐conferencing sessions with four participants each, and we modified the NGCT process by adding a post‐meeting analysis phase to account for the three separate meetings. All three sessions occurred within the span of one week in May 2024 and lasted for 2–3 hours, which is within the recommended 2–4 hours (Manera et al. 2019). Figure 1 outlines our modified NGCT process.

Each meeting began with an introductory presentation and setting meeting objectives. Participants were given a taxonomy developed from a single country (Porat‐Dahlerbruch et al. 2024), which included policy interventions mapped to broad categories across macro, meso, and micro levels. Participants were asked to suggest edits, revisions, and changes to any component of the single‐country taxonomy to internationalize it. We described “internationalizing” as enhancing applicability to one's country of expertise and other countries. We asked each group to develop its own taxonomy, stemming from the original single‐country taxonomy, without viewing feedback from the other groups. We encouraged participants to reflect on their past experiences with APN integration policy interventions, which helped capture experiences from when APN roles were not yet introduced, new, or less developed in their respective countries. This allowed us to capture perspectives on policy interventions intended for countries with less developed APN roles.

Next, the participants spent 20 minutes on silent generation (reading, reviewing, and making notes on the single‐country taxonomy). Then, each participant was given five minutes to express their initial ideas during the round‐robin session. The next part was open clarification and discussion. Although the participants discussed changes, one research team member took notes, and another made real‐time revisions to the taxonomy on “screen share” mode. At the end of each session, we conducted a consensus vote on the group's final taxonomy. Consensus was set at 70%, and only participants could vote (Belton et al. 2019).

### 3.4 Data Analysis

Analysis for traditional, single‐meeting NGCTs occurs during the meeting itself; in a sense, participants analyze the data together (Manera et al. 2019). Nonetheless, since this NGCT consisted of three separate groups, a post‐meeting analysis was necessary. As such, the meetings were recorded and transcribed. The research team reviewed the meeting transcript to ensure that all changes discussed during the meeting were made (McMillan et al. 2016). Two researchers, separately, analyzed the taxonomies from the three sessions and synthesized them into a single version. The researchers met to compare their versions and arrive at an agreement on a single version of the taxonomy. The synthesized taxonomy was sent to each participant via e‐mail for member checking (Motulsky 2021). Minor additional changes were requested, and the participants voted again over e‐mail on whether they agreed with the final taxonomy. A final 70% threshold was set (McMillan et al. 2016).

### 3.5 Trustworthiness

We undertook several measures to ensure trustworthiness based on recommendations from Ahmed (2024). We practiced reflexivity by documenting personal, interpersonal, methodological, and contextual thoughts and potential biases (Olmos‐Vega et al. 2022). We enhanced credibility through participant and investigator triangulation (Ahmed 2024). We ensured dependability by maintaining an audit trail of all our steps (Ahmed 2024).

### 3.6 Ethical Considerations

This study was approved by the Ben‐Gurion University of the Negev Human Subjects Research Committee (ME25122023). Informed consent was obtained. Participants were informed fully about the study's purpose, procedures, potential risks and benefits, and the right to withdraw at any time without penalty. Participants were informed that the nature of an NGCT presents the likely risk of revealing their identities to colleagues in the same session, though the research team did not reveal participant identities to members in the other NGCT sessions. In the transcripts, participants were assigned unique identification codes, and all data were stored securely to prevent unauthorized access. No identifying information was included in reports or publications.

## 4 Results

### 4.1 Participant Characteristics

Participant characteristics can be seen in Supplementary Material S1. Twelve experts from 10 countries participated—Canada, Chile, Ireland, Israel, New Zealand, Singapore, Tanzania, the United Kingdom, the United States, and Zimbabwe. Seven participants were academic experts, and five were practice experts. Among practice experts, three worked in large health systems and two in government.

### 4.2 International APN Integration Policy Intervention Taxonomy

**FIGURE 2:**
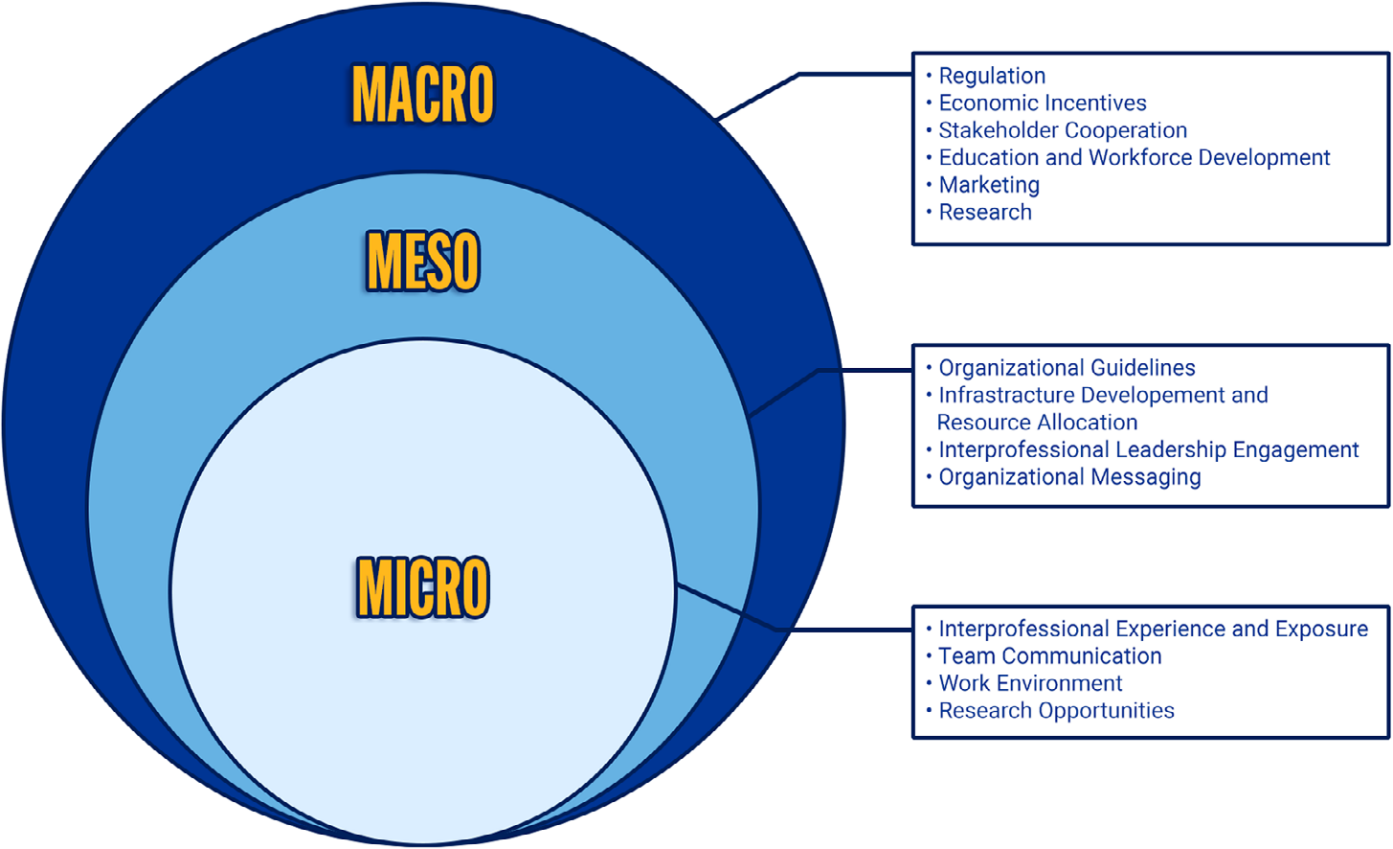
Illustration of the advanced practice nurse integration policy intervention taxonomy. There are three domains in the taxonomy—macro, meso, and micro. Macro represents the national, jurisdictional, regional, or international level. Meso represents the care organization level, such as a hospital, hospital system, or healthcare maintenance organization. Micro represents the point of service, such as a care team in a clinic or unit. Macro‐level policies affect meso‐level policies, which affect micro‐level policies. This “trickle‐down” effect is represented by the circles encompassing one another. The text boxes on the right show the policy intervention categories linked to each health system level.

Ninety‐two percent of participants agreed with the final taxonomy, which is illustrated in Figure 2. The three rings in Figure 2 represent the three health system levels at which policy interventions affect APN integration. Policy trickles down between the three health system levels—that is, the macro level affects the meso level, and the meso level affects the micro level. For instance, without clear scope of practice laws or regulations at the macro level, organizations may face legal uncertainty when developing guidelines for APN roles at the meso level. In turn, if meso‐level policy is not developed robustly, other care team members may be unsure how to work with APNs or what tasks can be allocated to them. As a result, APN practice is hindered, creating inefficiencies in the health system.

In the next sections, we summarize the intervention categories and explain how the interventions impact APN integration, grounded in the participants’ reasoning. Specific policy interventions within each category can be seen in Supplementary Material S2.

### 4.3 Macro‐Level Policy Interventions

The macro level encompassed national, jurisdictional, regional, and international interventions. There were 27 policy interventions across six macro‐level policy categories (Supplementary Table S2.1).

#### 4.3.1 Regulation

Participants described regulation as the governance and oversight of APNs at national and/or jurisdictional levels, depending on the health system. This category includes scope of practice, autonomy, licensure, and role titles, which all should align with the International Council of Nurses (2020, 2024) recommendations for APN roles and the *Global Strategic Plan*. Participants noted that *regulation* interventions exemplify “trickle‐down” APN policy from macro‐to‐meso levels. For example, without a clearly delineated scope of practice and APN role definition at the macro level, care organizations often are unsure how to develop policies to incorporate APNs and fear legal ramifications for permitting certain authorities, such as prescribing.

#### 4.3.2 Economic Incentives

The *economic incentives* category refers to national, jurisdictional, or regional initiatives promoting organizations to hire APNs and bedside nurses to seek APN education. Participants noted that economic incentives are key to sustainability—it is difficult to create buy‐in at the meso level when first introducing APN roles if macro‐level parties, such as government, do not subsidize organizations to pilot APN roles or do not compensate them for nurses spending time on higher education. One key example of *economic incentives* includes setting APN salaries and/or care payment structures at a professional rate that sufficiently creates a return on investment for APNs seeking the expanded role. Organizations, too, should be incentivized financially to recruit and retain APNs. Further, some health systems with more advanced APN integration have tried to incentivize organizations to promote high‐quality APN care by incentivizing positive APN‐linked patient outcomes.

#### 4.3.3 Stakeholder Cooperation

*Stakeholder cooperation* highlights another example of the macro‐to‐meso “trickle‐down” effect. The stance of national or regional interprofessional groups, namely national physician groups, tends to lead to supportive or antagonistic relationships between APNs and colleagues within a care organization. Organizational leaders making organizational policy decisions, such as chief medical officers or hospital directors, are influenced by messaging from the leadership in their respective professional groups. To address this concern, participants suggested coordinating integration efforts and planning with interprofessional groups, as opposed to driving efforts unilaterally through the nursing profession. Nevertheless, participants were clear that the APN profession should be self‐governed by nurses, as is typical with other professions.

#### 4.3.4 Education and Workforce Development

Many *education and workforce development* policy interventions center around pre‐ and post‐licensure policies promoting readiness for high‐quality, independent practice. Participants from countries with shorter and longer histories of APNs expressed that one of the most significant barriers to APN integration is a lack of clinical experience after graduation or expecting organizations to invest in APN clinical education at their first place of work. Organizations often do not have the funds to educate APNs after their licensure. Further, in countries with shorter histories of APN roles, organizational leadership is skeptical that APNs will provide a return on investment.

Some macro‐level *education and workforce development* interventions are more beneficial to countries newly introducing APN roles. For instance, easing international license transferability and establishing competency‐based education facilitate qualified international educators to teach in graduate‐level programs in countries recently establishing APN roles that lack the higher education infrastructure to educate APNs.

#### 4.3.5 Marketing

Often, the public and healthcare colleagues are unaware of APN roles and educational preparation. A lack of awareness can induce hesitancy for patients to receive APN care or for clinicians to work with APNs. Marketing campaigns can spread awareness about the APN role. Further, school‐age and college students should be exposed to the APN role and career mobility, thus attracting them to APN roles and growing the nursing workforce.

#### 4.3.6 Research

Macro‐level research efforts refer to larger‐scale research demonstrating APN care quality. Such research typically links APN care to patient outcomes, such as length of hospital stay, wait times, and hospitalizations. Developing this robust evidence base of APN care quality is one integration hurdle in health systems with long‐established and recently introduced APN roles. In each country, research should be conducted on APN‐linked outcomes, including access, utilization, quality, and cost. Current utilization of APNs across systems and sectors, including health and academic settings, should be evaluated. Nevertheless, participants discussed an existing paradox—countries with less nursing research infrastructure often exhibit less advanced APN integration, rendering it difficult to conduct robust research on APN‐linked outcomes.

The participants noted a need for an international synergy of research activities in two ways. First, participants cited the International Council of Nurses’ (2024, p. 8) *Global Strategic Plan*, calling for multinational research on the impact of broadening APN scope of practice. Participants believed that coordinated research efforts would help create a sustainable APN workforce across countries. Second, international research efforts should help push toward standardization of APN roles, like in medicine. Standardization would help clarify the scope of APN practice internationally, allow APNs to teach in countries with less developed APN workforces, and facilitate the international flow of APN human capital.

### 4.4 Meso‐Level Policy Interventions

The meso level included organization‐level interventions (e.g., hospital, hospital system, or healthcare maintenance organization). The meso‐level policy interventions included 22 actions across four categories (Supplementary Table S2.2).

#### 4.4.1 Organizational Guidelines

Care organizations are responsible for delineating guidelines for the APN role, in accordance with macro‐level regulation. Like macro‐level regulation, organizational guidelines should be clear, not limiting, and provide direction for managers, clinicians, and other employees. Ideally, organizations should create guidelines maximizing APN scope of practice and education. Participants from all three groups raised the notion that the key to developing *organizational guidelines* that promote the efficacious APN practice is including APNs in organizational leadership and decision‐making committees. Further, organizational guidelines should be evaluated and revised regularly, particularly when APNs are new to an organization.

#### 4.4.2 Infrastructure Development and Resource Allocation

Infrastructure and resources facilitate APN practice, according to the role outlined in an organization's guidelines. Though, participants noted that developing infrastructure takes time. For instance, participants recommended several policy interventions, such as forming an APN integration plan for all settings in an organization, establishing APN leadership and reporting structures, providing adequate support staff and resources for APNs, and establishing a leadership group. These interventions, alone, typically require years to develop. Participants agreed that in larger countries and countries with less centralized health systems, developing the infrastructure and efficiently allocating resources for APN integration requires more time.

#### 4.4.3 Interprofessional Leadership Engagement

Participants noted that *interprofessional leadership engagement* causes other health professional leaders to be exposed to the APN role and their educational preparation. The opinion of interprofessional leaders at the meso level often trickles down to their colleagues at the micro level, thus influencing opinions throughout a care organization. Accordingly, organizations should try to include APNs in events and communications with other care team members.

#### 4.4.4 Organizational Messaging

*Organizational messaging* refers to electronic or verbal communication from a care organization's leadership. Organizational messaging can be as broad as an information campaign about the APN role and authorities, though it can also be thought of as the detailed choice of diction surrounding APNs, such as phone scripts given to appointment schedulers who offer care from APNs. Organizational messaging can range from positive and prolific to unsupportive and unproductive. Participants identified organizational messaging as a factor influencing the culture surrounding working with APNs.

### 4.5 Micro‐Level Policy Interventions

The micro level included policy interventions at point of service (e.g., clinic or unit). Ten interventions at the micro level were distributed across four policy categories (Supplementary Table S2.3).

#### 4.5.1 Interprofessional Experience and Exposure

Participants in countries with divided hierarchies between health professions and/or with a greater presence of a patriarchy in society noted that experience and exposure were particularly pertinent; these participants said that healthcare workers in their countries tend to form judgment more quickly based on the health profession's “status” or the clinician's gender. As such, exposing the care team to APNs and their care can help temper judgments that may not be conducive to strong working relationships.

#### 4.5.2 Team Communication

In countries without well‐established non‐physician provider roles, physicians do not learn interprofessional communication with non‐physician providers. Further, APN programs often do not include formal education on team communication. As such, care teams should institute their own measures to promote communication. Examples include regular care team meetings or in‐service communication skill development.

#### 4.5.3 Work Environment

Research has established the role of the work environment in fostering collegiality, independent practice, and comfort in completing tasks. However, participants agreed that there is less existing guidance on how to develop work environments conducive to APN care. Participants noted a few potential options that have worked in their respective countries: (1) developing mentoring relationships among care team members, (2) ensuring administrative support, and (3) allocating time for professional clinical skill development (e.g., care team members teaching one another).

#### 4.5.4 Research Opportunities

A hallmark of APN practice is the ability to conduct research, improving care delivery at one's place of work. However, time and resources must be allocated to APNs to conduct quality improvement or implementation research projects. Allocating resources and encouraging implementation and evaluation of evidence‐based clinical practices ideally would improve care quality in their unit or clinic. Importantly, the emphasis on this intervention is in the APN's care setting, which contrasts with macro‐level research that focuses on larger‐scale research intended to demonstrate APN care quality or other outcomes.

## 5 Discussion

Through expert consensus, we developed the International APN Policy Intervention Taxonomy (Figure 2; Supplementary Material S2). The taxonomy consists of policy interventions to advance the integration of APNs at three health system levels (i.e., macro, meso, and micro), each of which targets different health system actors. At each level, specific policy interventions were mapped to broader categories. Macro‐level intervention categories include regulation, economic incentives, stakeholder cooperation, education and workforce development, marketing, and research. Meso‐level categories include organizational guidelines, infrastructure development and resource allocation, interprofessional leadership engagement, and organizational messaging. Micro‐level categories include interprofessional experience and exposure, team communication, work environment, and research opportunities. We provide a practical guide for applying this taxonomy to policy development in the implications section.

The results showed that APN integration can be visualized as a positive exponential function—introducing and advancing through the initial stages of APN integration takes time, though advances at an accelerating rate. This conclusion is grounded in several results. First, participants noted that developing infrastructure for APNs takes a significant amount of time in countries newly introducing APNs. Second, educating APNs at the recommended graduate level (International Council of Nurses 2020, 2024) requires licensed APNs to teach APN students, creating difficulty in initiating the first generation of APNs. Finally, participants noted a positive relationship between infrastructure to conduct APN‐linked outcomes research and APN integration; it is difficult to produce the initial robust evidence that APNs produce positive care outcomes. The policy interventions to address these paradoxical issues focus on utilizing APNs from countries with more advanced integration to counsel policymakers, assist in research infrastructure development, and educate the first generations of APNs (Bruce et al. 2017; Trinitas and Blood‐Siegfried 2023). However, educators from the home nations still need to teach APNs to provide population‐specific care (Trinitas and Blood‐Siegfried 2023).

The results captured some cultural nuances focused on health professional hierarchies and gender roles. Participants from some countries noted that the integration process is hindered when there is a greater presence of health system hierarchies, typically consisting of physicians providing orders to nurses, and nurses carrying out the orders (Kidd et al. 2023; Porat‐Dahlerbruch et al. 2023b). Further, countries with more prominent patriarchal structures tend to experience more difficulty establishing collegial care team–level relationships because APNs tend to be women, and physicians tend to be men (De Raeve et al. 2024; Porat‐Dahlerbruch et al. 2023a). The interventions addressing cultural nuances target the micro level, particularly exposure to and experience working with APNs (Miller et al. 2025; Taylor et al. 2021).

Policymakers in countries introducing APNs are expected to develop population‐specific, evidence‐based policies facilitating APN integration. However, again, this is a paradoxical task. Evaluating whether population‐specific policies are effective requires policy implementation. Applied here, APNs must first be introduced to evaluate integration policies. At that point, only ad hoc revisions to policy occur, which is known to create inefficiency and resource waste, risking the development of APNs in countries with limited resources (Bryant‐Lukosius et al. 2004; Porat‐Dahlerbruch et al. 2023a). The International APN Integration Policy Intervention Taxonomy presented in this study can be used to prevent resource waste associated with ad hoc policymaking by providing a broad framework of evidence‐based policy interventions that can be adapted and applied to specific settings at the outset. The key term here is “adapt.” That is, the broad interventions offered by this taxonomy can be *adapted* to specific settings and health system structures.

The policy interventions identified in this taxonomy are not groundbreaking, per se—the results mirror other international studies discussing policy options for improving the integration of APNs (De Raeve et al. 2024; Maier et al. 2017; Torrens et al. 2020). The novel aspect of this taxonomy, instead, is its broad applicability across countries and settings and its consumer‐friendly presentation. Prior studies tend to focus on countries with more developed APN roles because more data are readily available on the role (Porat‐Dahlerbruch et al. 2022). However, this leaves countries newly introducing APN roles with an insufficient evidence base for developing policy (Porat‐Dahlerbruch et al. 2023a). This study's taxonomy was developed with participants from countries with varying health system types, wealth, and stages of APN development, hence its broader applicability potential compared to prior studies. Nevertheless, it is important to note that this study did not include countries without any APN role, which limits the transferability of this taxonomy to countries without APNs.

### 5.1 Limitations

One critique of any consensus study is that sampling is non‐random, which raises questions about result generalizability (Belton et al. 2019). It has been argued that the goal of consensus studies is not to achieve generalizability but rather transferability (Drisko 2024). Transferability involves providing a detailed description of the phenomenon so that others can evaluate the extent to which the conclusions apply to other times, contexts, and populations. Additionally, participant composition influences NGCT studies. Participants are often selected based on convenience and specific criteria, rendering the sampling as non‐random (Belton et al. 2019). Although this approach can introduce bias, ensuring a heterogeneous sample can enhance transferability by incorporating diverse perspectives. We strived for heterogeneity in our sample to mitigate biased responses (Belton et al. 2019). In addition, although our study included 12 experts specializing in 10 countries, geographic representation is still limited. Further, all countries included in this study have somewhat of an established APN role, and there were no countries included without an APN role. For these two reasons, consumers of this taxonomy in countries without established APN roles should be cautious about transferability to their settings. Further work in countries without established APN roles could make this taxonomy more transferable to these audiences. Finally, although the international applicability of the taxonomy is a strength, it is also weakness—many actions may not be applicable to every setting. Nonetheless, irrelevant actions can be removed in a version of the taxonomy adapted to specific health systems, organizations, or care settings.

## 6 Conclusions and Recommendations

By incorporating insights of experts across the world, this taxonomy provides a comprehensive, globally adaptable framework for APN integration. The taxonomy can be applied at macro, meso, and/or micro levels. Implementing interventions from this taxonomy can enhance the effectiveness of APNs in the health system, and ultimately improve patient, system, and population outcomes.

## 7 Implications

The trichotomy of macro‐, meso‐, and micro‐health system levels separates the taxonomy's intended policy audience (Porat‐Dahlerbruch et al. 2025c). The macro‐level policy interventions can be applied by regulators, legislators, and national or regional professional stakeholders. Meso‐level policies could be used by organizational administrators, such as executives, managers, and organizational leaders. The micro‐level policy interventions can be consumed by APNs, physicians, other clinicians, and clinic or unit managers.

**FIGURE 3:**
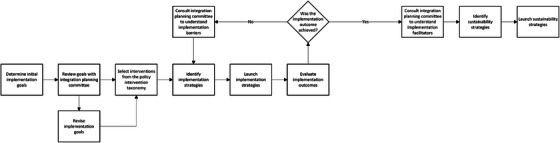
Steps for applying the International Advanced Practice Nurse Policy Intervention Taxonomy. These steps were adapted from a synthesis of instructions for policy development and research from three implementation science frameworks: (1) Consolidated Framework for Implementation Research (Damschroder et al. 2022), (2) Behavior Change Wheel (Michie et al. 2011), and (3) Context and Implementation of Complex Interventions framework (Pfadenhauer et al. 2017).

Applying a broad taxonomy to specific settings and populations, as suggested here, is common in implementation science. Examples include the Consolidated Framework for Implementation Research (Damschroder et al. 2022), the Behavior Change Wheel (Michie et al. 2011), and the Context and Implementation of Complex Interventions Framework (Pfadenhauer et al. 2017). The authors of these works provide instructions for applying the frameworks to achieve desired outcomes, which have been used for policy development at macro, meso, and micro levels—e.g., human papillomavirus vaccine distribution and uptake across Mozambique (Soi et al. 2018) and health technology use assessments in care organizations and clinics (Esmail et al. 2021). We posit that policy decision‐makers can apply these same steps to the International APN Integration Policy Intervention Taxonomy—see the steps outlined in Figure 3. When policy decision‐makers implement the policy interventions from this taxonomy, researchers or the policy decision‐makers themselves should publish their selected implementation strategies and outcomes. Once enough evaluations are published, a knowledge synthesis, such as an integrative or systematic review, can be conducted to inform other policy decision‐makers about the implementation strategies used when applying the intervention taxonomy.

## Author Contributions

Conceptualization: JPD. Methodology: JPD. Software: JPD. Formal analysis: TM and JPD. Investigation: JPD. Data curation: JPD. Writing—original draft: JPD and TM. Writing—review and editing: JB, ME, and RC. Visualization: JPD and JB. Resources: JPD. Project administration: JPD. Supervision: JPD. Validation: JB, ME, and RC.

## Conflicts of Interest

No conflict of interest has been declared by the authors.

## Supporting information




**Table S1**: Participant Characteristics.

**Table S2.1**: Macro‐Level Policy Categories and Interventions to Advance the Integration of Advanced Practice Nurses into Health Systems.
**Table S2.2**: Meso‐Level Policy Categories and Interventions to Advance the Integration of Advanced Practice Nurses into Health Systems.
**Table S2.3**: Micro‐Level Policy Categories and Interventions to Advance the Integration of Advanced Practice Nurses into Health Systems.
