# Supplementary material for: The International Advanced Practice Nurse Integration Policy Intervention Taxonomy: A 10‐Country Nominal Group Consensus Technique Study
Source: Int Nurs Rev. 2025 Aug 12;72(3):e70096. doi: 10.1111/inr.70096 (PMC12344393; doi:10.1111/inr.70096)
Supplement: Supplementary file 1 — Table S1: Participant Characteristics. [file INR-72-0-s002.docx]

**SUPPLEMENTARY MATERIAL 1:** Participant Characteristics

**Table S1: Participant Characteristics**

| **Characteristic** | **No., %** |
| --- | --- |
| **Total** | 12 (100%) |
| **Location** |  |
| Canada | 1 (8%) |
| Chile | 1 (8%) |
| Ireland | 1 (8%) |
| Israel | 1 (8%) |
| New Zealand | 1 (8%) |
| Singapore | 1 (8%) |
| Tanzania | 1 (8%) |
| United Kingdom | 1 (8%) |
| United States | 3 (25%) |
| Zimbabwe | 1 (8%) |
| **Work Setting** |  |
| Academia | 7 (58%) |
| Healthcare Setting | 3 (25%) |
| Government | 2 (17%) |

**Table S1 Caption:** This table displays the characteristics of experts who participated in the nominal group consensus meeting. The “location” category represents the country in which their advanced practice nurse integration expertise is focused.
