# Supplementary material for: The International Advanced Practice Nurse Integration Policy Intervention Taxonomy: A 10‐Country Nominal Group Consensus Technique Study
Source: Int Nurs Rev. 2025 Aug 12;72(3):e70096. doi: 10.1111/inr.70096 (PMC12344393; doi:10.1111/inr.70096)
Supplement: Supplementary file 2 — Table S2.1: Macro‐Level Policy Categories and Interventions to Advance the Integration of Advanced Practice Nurses into Health Systems. Table S2.2: Meso‐Level Policy Categories and Interventions to Advance the Integration of Advanced Practice Nurses into Health Systems. Table S2.3: Micro‐Level Policy Categories and Interventions to Advance the Integration of Advanced Practice Nurses into Health Systems. [file INR-72-0-s001.docx]

**SUPPLEMENTARY MATERIAL 2:** Policy Categories and Interventions by Health System Level

**Table S2.1:** Macro-Level Policy Categories and Interventions to Advance the Integration of Advanced Practice Nurses into Health Systems

| Policy Category | Interventions |
| --- | --- |
| Regulation | - Clearly define the APN role across systems. - Define APN scope of practice that includes autonomous practice. - Differentiate the APN discipline. |
| Economic Incentives | - Reimbursement and salaries for APN care at a professional rate. - Provide economic incentives for organizations to recruit and retain APNs and promote positive work environment. - Subsidize costs of sending nurses for APN education (e.g., time off payments). - Incentivize organizations for APN contributions to the system and patient care. |
| Stakeholder Cooperation | - Coordinate APN integration efforts with national interprofessional groups. - Promote APN self-regulation on parity with other professions. - Work with health professional, hospital, and care organization stakeholder groups when creating a national plan for integrating APNs. - Communicate APN scope of practice to relevant stakeholders and partners. |
| Education and Workforce Development | - Align and monitor the number of APNs educated in accordance with workforce position availability and population needs across specialties. - Create and fund more positions for APNs. - Transition into standardized competency-based education. - Fund transition to practice programs. - Incentivize preceptorship programs. - Subsidize universities to open APN degree programs. - Establish APN program curricula based on existing international evidence. - Train faculty with relevant education according to the International Council of Nurses (2024) *Global Strategic Plan*. |
| Marketing | - Initiate an advertising campaign to inform the public of the new APN role. - Begin an information campaign on the APN role targeted at health professional colleagues. - Expose high school and college students to the APN role and career mobility. - Differentiate APNs from other healthcare services in marketing campaigns. |
| Research | - Establish international cohesion through research so that there can be a gold standard understanding of the APN role. - Conduct research on APN-linked health system and patient outcomes (access utilization quality and cost). - Evaluate the current utilization of APNs across the systems and sectors including health and academic settings. - Synergize research efforts between international, national and regional levels |

**Table S2.1 Caption:** The macro level refers to the national, jurisdictional, regional, or international level. APN=advanced practice nurse

**Table S2.2:** Meso-Level Policy Categories and Interventions to Advance the Integration of Advanced Practice Nurses into Health Systems

| Policy Category | Interventions |
| --- | --- |
| Organizational Guidelines | - Clearly define the APN role in both clinical and leadership domains. - Clearly describe APN scope of practice within the organization. - Create an organizational care model that includes APNs and recognizes full scope of APN care. - Promote equitable APN involvement in leadership, shared decision making and policy making. |
| Infrastructure Development & Resource Allocation | - Outline a formal plan for APN integration. - Integrate APNs across all settings. - Consider establishing APN leadership and reporting structures. - Provide adequate support staff and resources to APNs. - Establish a leadership group for constant policy development for the APN role with representation from all organizational stakeholder groups. - Clearly define APN pay schedules and salary structures in parity with other health care professionals. - Systematically assess APN integration progress, communicate findings internally & update policies accordingly. - Assure data and technology infrastructure to APNs that promotes full access. - Develop onboarding programs and ongoing support for APNs. - Include role title, degrees, & certifications after APN names. - Create data infrastructure clearly attributing APN services to patient and system outcomes. - Encourage evidence-based practices to promote recruitment and retention. |
| Interprofessional Leadership Engagement | - Host interprofessional workshops and seminars with all providers. - Promote interprofessional decision making. - Promote interprofessional educational experiences. |
| Organizational Messaging | - Communicate the APN role, education/training, and scope of practice to clinicians throughout the integration process. - Disseminate messaging supportive of APNs to the organization’s employees. - Allocate time to educate organizational leaders on APN care benefits. |

**Table S2.2 Caption:** The meso level refers to the care organization level, such as a hospital, hospital system, or healthcare maintenance organization. APN=advanced practice nurse.

**Table S2.3:** Micro-Level Policy Categories and Interventions to Advance the Integration of Advanced Practice Nurses into Health Systems

| Policy Category | Interventions |
| --- | --- |
| Interprofessional Experience & Exposure | - Demonstrate APN holistic care and value to the care team. - Assure infrastructure that health care team members are aware of APN value and holistic care methods. |
| Team Communication | - Institute regular care team meetings to promote consistent communication. - Educate care teams on interprofessional communication skills. |
| Work Environment | - Encourage appropriate mentoring relationships within the care team. - Provide administrative support including infrastructure, resources and professional skill development. - Ensure that patients and families are aware of the APN role. |
| Research Opportunities | - Promote APN-led quality improvement research. - Encourage implementation of evidence-based practice. - Advocate for constant evaluation of current clinical practices. |

**Table S2.3 Caption:** The micro level refers to the point-of-service, such as a care team in a clinic or unit. APN=advanced practice nurse.
